# Impact of *Curcuma longa L.* extract supplementation on the gut microbiota of hemodialysis patients

**DOI:** 10.1590/2175-8239-JBN-2025-0032en

**Published:** 2025-12-12

**Authors:** Livia Alvarenga, Ludmila Cardozo, Júnia Schultz, Fluvio Modolon, Alexandre Rosado, Denise Mafra

**Affiliations:** 1 Universidade Federal do Rio de Janeiro, Programa de Pós-Graduação em Ciências Biológicas – Fisiologia, Rio de Janeiro, RJ, Brazil.; 2 Universidade Federal Fluminense, Programa de Pós-Graduação em Ciências da Nutrição, Niterói, RJ, Brazil.; 3 Universidade Federal Fluminense, Programa de Pós-Graduação em Ciências Cardiovasculares, Niterói, RJ, Brazil.; 4 King Abdullah University of Science and Technology, Biological and Environmental Science and Engineering Division, Thuwal, Makkah, Saudi Arabia.; 5 Universidade de São Paulo, Instituto Oceanográfico, São Paulo, SP, Brazil.

**Keywords:** Curcumin, Curcuma longa, Gut microbiota, Hemodialysis, Chronic Kidney Disease

## Abstract

**Introduction:**

The impact of curcumin on the gut microbiota of chronic kidney disease (CKD) patients is not well known. The aim of this study was to evaluate the effect of *Curcuma longa L.* on the gut microbiota of CKD patients undergoing hemodialysis (HD).

**Methods:**

This was a secondary analysis of data from a randomized, double-blind, placebo-controlled trial. Patients received 100 mL of orange juice, 12 grams of carrot, and 2.5 grams of *Curcuma longa L.* three times a week after the HD session (Curcuma group) or the same juice without added curcumin (control group) for 12 weeks. The fecal microbiota composition was estimated using short-read sequencing of the V4 region of the 16S rRNA gene on the Illumina platform.

**Results:**

Eleven patients participated in this study, five in the curcumin group (66.7% male, 59 ± 16.7 years old, HD vintage of 97 ± 62.6 months, BMI 25.3 ± 2.9 kg/m^2^) and six in the control group (60% male, 57.5 ± 12.5 years old, HD vintage of 48.3 ± 32.2 months, BMI 25.2 ± 3.1 kg/m^2^). Supplementation with *Curcuma longa L.* extract did not modify alpha biodiversity or the taxonomic composition of individuals at the phylum, family, and genus levels.

**Conclusion:**

Supplementation with 2.5 g of *Curcuma longa L.* extract three times per week for 12 weeks was inefficient in modulating the gut microbiota of CKD patients undergoing HD. These results should be interpreted taking into account the small sample size, and future studies with larger cohorts are encouraged.

## Introduction

Gut dysbiosis is the imbalance between intestinal bacteria, consequently impairing their metabolic activity, and this condition is commonly found in patients with chronic kidney disease (CKD)^1,2,3^. Experimental and clinical studies have demonstrated that patients with CKD have gut dysbiosis with high production of toxic metabolites by pathobionts and low abundance of beneficial species involved in the production of short-chain fatty acids^4,5^.

The loss of kidney function is associated with increased urea secretion in the gastrointestinal tract and the generation of large amounts of ammonia from urea hydrolysis by intestinal bacteria^6^. The accumulation of ammonia increases intestinal pH, severely irritates the mucosa, affects the growth of commensal bacteria, and maintains the dysbiotic state^6,7^.

Furthermore, patients with CKD consume less fiber, which, along with using phosphate binders, decreases colonic transit time in the gut microbiota^8^. Consequently, uremic solutes produced during fermentation by the intestinal microbiota accumulate in CKD patients^1,7^.

Gut dysbiosis in CKD patients accelerates disease progression, causes complications and systemic inflammation, and raises mortality rates. Therefore, nutritional strategies have been proposed to address gut dysbiosis in these patients^9,10^. *Curcuma longa L.* is rich in bioactive compounds called curcuminoids and has been the target of recent studies with patients with CKD, demonstrating crucial anti-inflammatory properties^11,12^ and beneficial effects on gut health^13,14^.

The hypothesis of the present pilot study was that supplementation with *Curcuma longa L*. extract (95% curcumin) modulates the microbiota of patients with CKD undergoing hemodialysis. This study aimed to evaluate the effect of *Curcuma longa L.* (95% curcumin) on the intestinal microbiota profile of hemodialysis (HD) patients.

## Methods

A secondary analysis of data from a longitudinal, randomized, and double-blind research in patients undergoing HD was conducted^11^. No formal sample size calculation was conducted for the outcomes assessed in this sub-analysis. The Faculty of Medicine/UFF Ethics Committee approved the study, registered under number 2.346.933 and on ClinicalTrials.gov with the identifier NCT 03475017.

### Subjects

Patients aged 18 years or more and undergoing HD (via an arteriovenous fistula) for over six months were included in the study. The exclusion criteria were pregnant women, smokers, patients who had taken antibiotics within the past three months, individuals using antioxidant supplements or regularly consuming turmeric, and those with autoimmune and infectious diseases, cancer, liver diseases, and AIDS. Medications used before the study were maintained throughout the study.

### Experimental Design

Patients were randomized in a double-blind manner into two groups: the curcumin group, which received 100 mL of orange juice with 12 grams of carrot and 2.5 grams of turmeric (95% curcumin) three times a week after the HD session for 12 weeks, and the control group, which received the same juice without curcumin.

The orange and carrot juice used as the vehicle for curcumin administration was standardized, and its nutritional composition was previously described by Alvarenga et al.^11^. Each 100 mL portion contained approximately 98 kcal, 22.6 g carbohydrates, 0.2 g lipids, 1.0 g protein, 3.6 g fiber, 38.6 mg sodium, 44.0 mg phosphorus, and 323.0 mg potassium^11^. The *Curcuma longa L.* extract used in this trial was previously analyzed by Alvarenga et al.^15^ using HPLC-DAD. The extract contained 75.31 ± 2.0% curcumin, 18.42 ± 0.6% demethoxycurcumin, and 5.19 ± 0.2% bisdemethoxycurcumin, totaling 98.92 ± 2.8% w/w of total curcuminoids^15^.

### Food Intake Analysis and BMI Assessment

Food consumption was assessed before and after the intervention using the 24-hour food recall method and calculated using the NutWin^®^ software (Alvarenga et al.^11^). Body mass index (BMI) was calculated as body weight (kg) divided by squared height (m).

### Blood Collection and Biochemical Analysis

Routine biochemical exams, such as albumin, phosphorus, calcium, potassium, urea, parathyroid hormone (PTH), and hemoglobin, were obtained from medical records. Serum levels of high-sensitivity C-reactive protein (hs-CRP), total cholesterol, triglycerides, and c-HDL were determined using Bioclin BS-120 Chemistry Analyzer. Low-density lipoprotein (LDL) cholesterol was calculated using the Friedewald equation.

### Gut Microbiota Sequencing and Analysis

For the gut microbiota analysis, patients received sterile stool collection tubes and detailed instructions on how to collect samples. Total DNA extraction was performed using the Quick-DNA Fecal/Soil Microbe DNA Miniprep Kit from Zymo Research, following the manufacturer’s instructions. The DNA quality and quantity were determined by spectrophotometric quantification using the NanoDrop 2000 (Thermo Fisher Scientific)^16^.

The 16S rRNA gene (V4 region) was amplified by PCR using the universal primers 515F (5’-GTGYCAGCMGCCGCGGTAA-3’) and 806R (5’-GGACTACNVGGGTWTCTAAT-3’), appended with universal Illumina tags. A thermocycling of 3 min initial denaturation at 94°C followed by 32 cycles of 45 s at 94°C, 1 min at 50 °C, and 90 s at 72 °C, with a final extension step of 10 min at 72°C was conducted to generate the amplicons. The resulting amplicons were then barcoded, pooled, and sequenced with the Illumina NovaSeq PE250 platform, following the manufacturer’s instructions at Novogene (California, USA). In total, 22 samples from HD patients were amplicon-sequenced.

The 16S rRNA gene amplicon sequencing reads were pre-processed employing the USEARCH (v.11) pipeline (Edgar, 2010^17^). The resulting sequences were denoised into Zero-radius Operational Taxonomy Units (zOTUs), which included pooling samples while keeping unique sample tags in the fastq headers, followed by assembly/merging paired-end reads, trimming primers, and quality filtering. Additionally, abundances were calculated for unique sequences and clustering of zOTUs at 97% identity, followed by a denoising step to filter chimeras to obtain a zOTUs table. Feature, taxonomy, and metadata tables were exported as phyloseq objects for further analysis in RStudio (v. 2023.06.0+421).

To allow comparison on an equal basis between the studied groups, data were rarefied for downstream comparison analyses. Rarefied data generated alphadiversity indexes (observed number of zOTU and Shannon diversity). Raw zOTU data were normalized with DESeq2 and used to calculate weighted Unifrac distance using the phyloseq package implemented in R. Permutational multivariate analysis of variance (PERMANOVA) was performed on the data matrix to compare the structure or composition of the microbial communities. Permutational multivariate analysis of dispersion (PERMDISP) was also performed using the betadisper function (implemented in vegan 2.6–4) with 999 permutations. The package DESeq2 was used to evaluate the differential relative read abundances between sample groups. Plots and graphs were generated with the package ggplot2 (v.3.4.0).

### Statistical Analyses

The Shapiro-Wilk test was used to verify the distribution of the sample variables. For comparisons between groups, the Student’s t-test was used for samples with symmetric distribution and the Mann-Whitney test was used for variables with asymmetric distribution. For before-and-after comparisons within the same group, the paired Student’s t-test was used for variables with symmetric distribution, and the Wilcoxon test was used for variables with asymmetric distribution. The significance level was set at less than 5% (p < 0.05). Sample characteristic data are expressed as absolute frequencies for categorical variables and mean (standard deviation) or median (interquartile range) for quantitative variables. Statistical analyses were performed using SPSS 23.0 (SPSS, Inc., Chicago, IL, USA).

## Results

**Figure 1:**
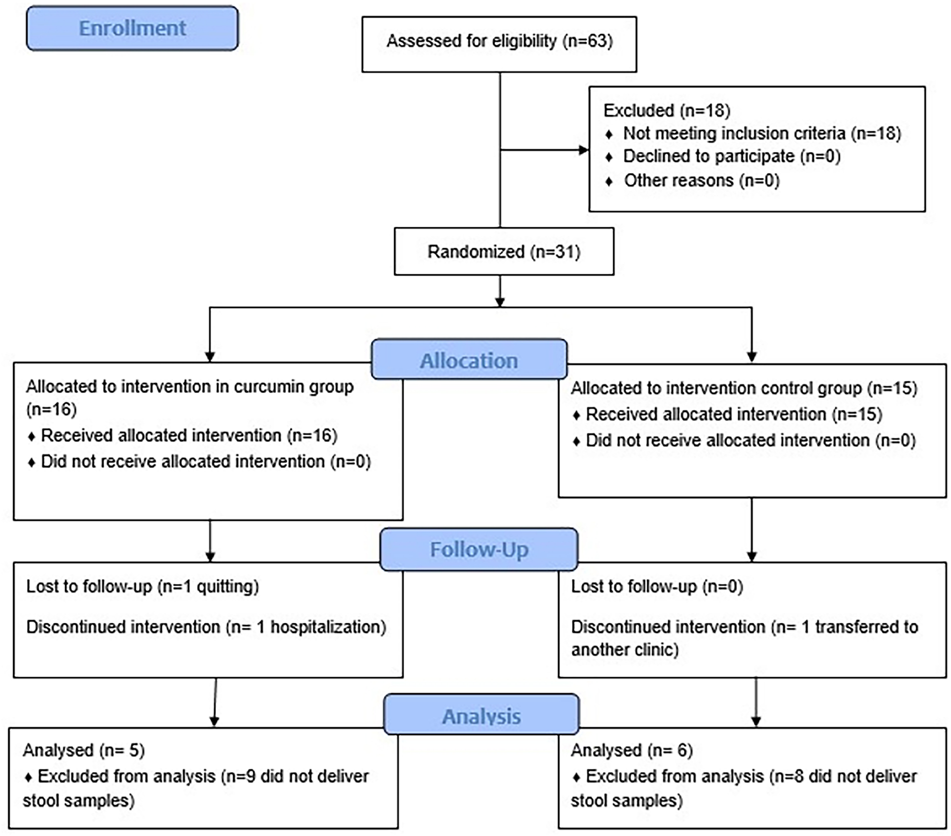
CONSORT flowchart of the study.

A total of 11 subjects were included in this study (Figure 1) (58.3% male, 58.2 ± 14.1 years old, K/tV 1.4 ± 0.1, time on hemodialysis of 70.4 ± 52.3 months, and BMI of 25.3 ± 2.9 kg/m^2^). They were divided into two groups, five in the curcumin group (66.7% male, 59 ± 16.7 years old, HD vintage of 97 ± 62.6 months, BMI 25.3 ± 2.9 kg/m^2^) and six in the control group (60% male, 57.5 ± 12.5 years old, HD vintage of 48.3 ± 32.2 months, BMI 25.2 ± 3.1 kg/m^2^).

The biochemical parameters and food intake characteristics before and after the intervention are shown in Table 1. At baseline, there were no statistically significant differences between the curcumin and control groups in any of the analyzed variables. In the curcumin group, a significant reduction was observed in total cholesterol, HDL, and LDL cholesterol. In the control group, there was also a significant reduction in LDL, along with a significant increase in serum calcium and PTH levels.

**Table 1:** Baseline characteristics of the patients with CKD undergoing hemodialysis

Parameters	Overall (n = 11)	Curcumin before (n = 5)	Curcumin after (n = 5)	p-values^a^	Control before (n = 6)	Control after (n = 6)	p-values^b^	p-values^c^	p-values^d^
Routine tests	
TC (mg/dL)	161.9 ± 32.4	164.6 ± 25.3	139.6 ± 19.8	**0.01**	159.7 ± 39.7	158.4 ± 54.2	0.06	0.82	0.45
Triglycerides (mg/dL)	127.2 ± 49.2	104.8 ± 30.7	115 ± 55.7	0.19	145.8 ± 56.4	188.6 ± 121.7	0.06	0.18	0.21
HDL (mg/dL)	34.3 ± 10.1	42.4 ± 7.4	39 ± 8.2	0.06	27.6 ± 6.4	37 ± 8.1	0.67	0.08	0.69
LDL (mg/dL)	102.4 ± 29.5	102.2 ± 28.9	77.6 ± 17	**0.01**	104.4 ± 32.6	83.7 ± 54.5	**0.01**	0.91	0.80
Calcium (mg/dL)	8.9 ± 0.6	8.8 ± 0.4	8.6 ± 0.6	0.37	8.1 ± 0.7	8.7 ± 0.6	**0.01**	0.92	0.68
Phosphorus (mg/dL)	4.8 ± 0.8	4.5 ± 0.7	5.1 ± 0.7	0.28	5.2 ± 0.9	5.7 ± 0.9	0.08	0.20	0.27
Albumin (mg/dL)	3.6 ± 0.2	3.6 ± 0.1	3.6 ± 0.2	0.53	3.6 ± 0.3	3.7 ± 0.2	0.54	1.00	0.45
Potassium (mg/dL)	4.7 ± 0.3	4.8 ± 0.2	4.9 ± 0.2	0.54	4.6 ± 0.5	4.7 ± 0.3	0.49	0.70	0.10
Hemoglobin (mg/dL)	11.06 ± 1.2	11.6 ± 2.4	10.9 ± 2.1	0.26	10.5 ± 1.4	11.8 ± 2.4	0.06	0.35	0.52
PTH (mg/dL)	560 (435.7–1414.8)	1027.5 (460.6–1856)	1075 (341–1846)	0.07	432.5 (208.3–1599.6)	899 (269–2640)	**0.01**	0.24	0.73
hsCRP (mg/L)	3.4 (2.5–11.5)	5.8 (1.4–13.1)	4.7 (0.2–16.6)	0.66	5.5 (0.1–16.2)	2.8 (0.7– 6.7)	0.58	0.58	0.56
Dietary intake	
Energy (Kcal/kg/day)	22.1 ± 6.9	21.3 ± 8.6	21.1 ± 11.8	0.16	22.8 ± 6.0	23.1 ± 9.7	0.34	0.74	0.76
Carbohydrate (g/day)	220 ± 74.4	111.6 ± 67.8	211.5 ± 99	0.33	227.2 ± 85.1	274.7 ± 146.5	0.47	0.75	0.41
Protein (g/kg/day)	1.0 ± 0.4	0.9 ± 0.5	1.1 ± 0.6	0.17	1.05 ± 0.3	1.1 ± 0.6	0.27	0.76	0.96
Lipid (g/day)	34.7 ± 12.9	30.6 ± 17	24.7 ± 11.4	0.12	38.0 ± 7	31.4 ± 18.3	0.78	0.37	0.48
Phosphorus (mg/day)	923.8 (715–1242.1)	819.6 (316.6–1323)	779 (323–1444)	0.34	1201 (732–1490)	1169 (602–1956)	0.15	0.24	0.19
Potassium (mg/day)	1627 (1373–2492)	1529 (1030–2028)	1663 (456–2669)	**0.03**	1841 (1241–3296)	1730 (878–2432)	0.53	0.17	0.88

Abbreviations – TC: total cholesterol; PTH: parathormone; HDL: high-density lipoprotein; LDL: low-density lipoprotein; hsCRP: high-sensitivity C-reactive protein.

Notes – Data are presented as mean (standard deviation) or median (interquartile range) for quantitative variable.

^a^Before and after comparison in the curcumin group.

^b^Before and after comparison in the control group.

^c^Baseline comparison between groups.

^d^Comparison between groups at the end of the intervention.

**Figure 2:**
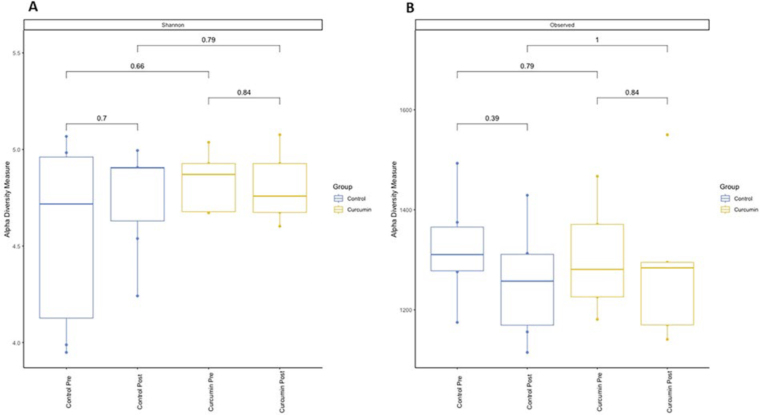
Difference in biodiversity before and after the intervention with curcumin. Bacterial ecological diversity was evaluated between the control group (blue) and the curcumin group (yellow). (A) Alpha-diversity analysis with Shannon index. (B) Analysis of alpha-diversity with observed species (OTU count).

**Figure 3:**
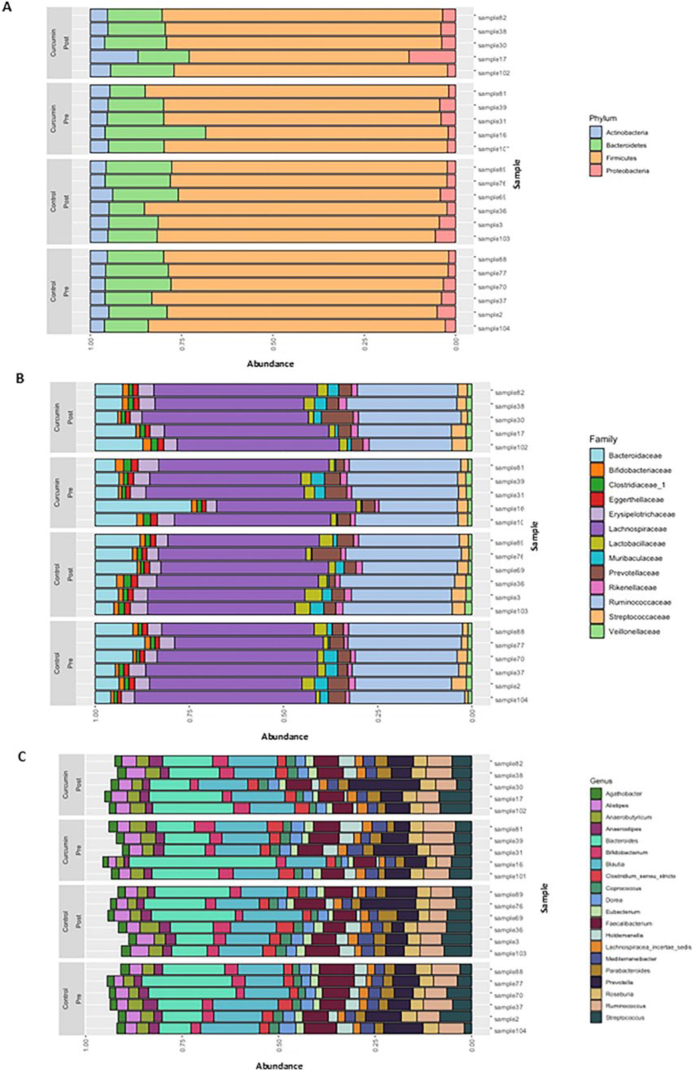
Taxonomic composition of individuals in the control and curcumin groups, before and after the intervention. Bar graphs of mean microbial relative abundances at the level of phylum (A), family (B), and genus (C) for individuals in the control group before (Pre) and after (Post) the intervention and individuals in the curcumin group before (Pre) and after intervention (Post).

There was no statistical difference in the gut microbiota biodiversity of HD patients, as measured by the Shannon index (Figure 2A), and observed species (OTU count) (Figure 2B) between groups (control and curcumin) and intervention period (Pre and Post). Also, there was no difference in the taxonomic composition between groups at the level of phylum (Figure 3A), family (Figure 3B), and genus (Figure 3C).

## Discussion

In the present study, curcumin supplementation did not change the diversity or the taxonomic structure of the gut microbiota over the 12 weeks of study in patients with CKD undergoing HD. This is the first study to examine the effect of curcumin on the gut microbiota composition in HD patients.

In a study involving non-dialysis CKD patients, supplementation with 1000 mg of *Curcuma longa L.* extract for 6 months effectively modulated the gut microbiota by reducing *Escherichia-Shigella* and increasing *Lachnoclostridium*. At the family level, *Lactobacillaceae* species increased after 3 months supplementation^13^. Other studies have demonstrated the effectiveness of *Curcuma longa L*. extract supplementation in decreasing uremic toxins produced by the gut microbiota in patients with CKD; however, the role of curcumin in influencing the gut microbiota has not been investigated. Salarolli et al.^14^, in a randomized, double-blind trial involving 28 HD patients, found that supplementation with 100 mL of orange juice containing 12 g of carrots and 2.5 g of turmeric three times a week for three months reduced plasma p-cresyl sulfate (pCS) concentrations. Reis et al.^18^ showed that supplementing with 1500 mg of *Curcuma longa L*. extract for twelve weeks indicated a trend toward lowering plasma pCS levels (p = 0.07) in peritoneal dialysis patients.

Curcuminoids, including curcumin, can be metabolized by the gut microbiota^18^ through a propsed hydroxylation reaction process, a common microbial metabolic step, forming the metabolites hydroxycurcumin and dihydrocurcumin, which modulate the gut microbiota^19,20^. Moreover, other metabolic pathways were identified, demonstrating that curcumin can be metabolized by the gut microbiota through reduction, methylation, demethoxylation, and acetylation processes, resulting in the formation of tetrahydrocurcumin, dihydroferulic acid, and 1-(4-hydroxy-3-methoxyphenyl)-2-propanol^20,21^. Bacteria probably involved the biotransformation and degradation of the compound include *E. coli*, *E. fergusonii*, *Blautia* sp., *Bifidobacterium*, *Lactobacillus*, *Enterococcus faecalis*, *Pichia anomala*, and *Bacillus megaterium* ^20,21,22^.

Therefore, the gut microbiota significantly influences the biotransformation of curcumin, and its metabolites produced by the gut microbiota also play a promising role in preventing or treating many diseases^22^. However, depending on its formulation, a greater microbial degradation of the original compounds might not occur^19^. The phospholipid formulation leads to greater microbial degradation of the parent compounds than the natural curcumin extract. In other words, the microbial biotransformation process is more efficient than with the simple curcuminoid extract^19^. This provides an insight into the ideal curcumin formulation for gut microbiota modulation. In addition, individual differences in microbiota compositions may cause different biotransformations of curcumin^22^, which may affect its benefits. This factor could transcend our findings in the present study.

Gut microbiota composition is influenced by multiple factors, including diet, medications, stress, physical activity, and environmental exposures. In the present study, dietary intake was assessed and taken into account when interpreting microbiota results, aligning with the increasing evidence that diet is a key factor in microbiota composition, especially in patients with CKD^23^. However, the other factors were not evaluated, and their potential influence on gut microbial variability cannot be ruled out. As highlighted by Van Hul et al.^24^, defining a “healthy microbiome” remains a complex challenge due to the interaction between host, lifestyle, and environmental factors.

Although the primary focus of this study was the gut microbiota, secondary analyses showed a significant decrease in total and LDL cholesterol levels in patients taking curcumin. These results align with previous evidence indicating the lipid-lowering effects of *Curcuma longa L.* extract. Alvarenga et al.^15^ reported a trend toward lower triglycerides in HD patients taking curcumin supplements, supporting its potential as an adjunctive approach for managing lipid metabolism. The mechanisms may include decreased intestinal cholesterol absorption by inhibiting the Niemann-Pick C1-like 1 (NPC1L1) transporter, increasing hepatic LDL receptors, and regulating enzymes involved in lipid synthesis, such as 3-hydroxy-3-methylglutaryl-CoA (HMG-CoA) reductase and fatty acid synthase^25,26^.

The findings of this pilot study should be interpreted in light of some limitations, particularly the small sample size, which substantially limits the statistical power for detecting differences in the microbiota. Therefore, the results should be considered exploratory and hypothesis-generating. Although the curcumin extract used in this study was standardized to 95% curcuminoids, a formulation commonly employed to ensure consistency and improve bioavailability compared to crude turmeric, curcumin’s limited systemic absorption remains a known limitation. In line with this, future studies may benefit from using more advanced delivery systems, such as co-administration with piperine, phospholipid complexes, or nanoparticulated forms, which have demonstrated superior pharmacokinetic profiles.

The absence of detectable microbiota modulation may stem from the complexity of the intestinal ecosystem and the study’s relatively short duration (12 weeks), which may have been insufficient to reveal the effects of curcumin on the microbiota. While 16S rRNA amplicon sequencing provides insight into the dominant members of the microbial community and their taxonomic resolution, it offers limited information about lower taxonomic levels. These levels may include rare species or community members whose variations, though significant, might not be captured through this method. Future studies should consider the use of shotgun metagenomic sequencing, which allows for deeper taxonomic resolution and direct assessment of functional microbial pathways, thereby overcoming the limitations of 16S rRNA profiling. In addition, future studies could benefit from long-term results of varying dosages of curcumin on the microbiota and explore its effects on broader CKD outcomes, including cardiovascular health, kidney function, and patients’ quality of life.

## Conclusion

Supplementation with 2.5 g *Curcuma longa L*. extract (95% curcumin) three times a week for 12 weeks was inefficient in modulating the gut microbiota of patients with CKD on HD. Future studies with larger cohorts are encouraged to further explore the potential effects of curcumin on the gut microbiota in this population.

## Data Availability

The full dataset supporting the findings of this study is available upon request from the corresponding author, Dr. Livia Alvarenga. The dataset is not publicly available due to ethical concerns.
