# Supplementary material for: Impact of Curcuma longa L. extract supplementation on the gut microbiota of hemodialysis patients
Source: J Bras Nefrol. 2025 Dec 12;48(1):e20250032. doi: 10.1590/2175-8239-JBN-2025-0032en (PMC12704657; doi:10.1590/2175-8239-JBN-2025-0032en)
Supplement: Translation

# Translation: Impacto da suplementação com extrato de Curcuma longa L. na microbiota intestinal de pacientes em hemodiálise

**DOI:** 10.1590/2175-8239-jbn-2025-0032pt

**Authors:** Livia Alvarenga, Ludmila Cardozo, Júnia Schultz, Fluvio Modolon, Alexandre Rosado, Denise Mafra

**Affiliations:** 1 Universidade Federal do Rio de Janeiro, Programa de Pós-Graduação em Ciências Biológicas – Fisiologia, Rio de Janeiro, RJ, Brasil.; 2 Universidade Federal Fluminense, Programa de Pós-Graduação em Ciências da Nutrição, Niterói, RJ, Brasil.; 3 Universidade Federal Fluminense, Programa de Pós-Graduação em Ciências Cardiovasculares, Niterói, RJ, Brasil.; 4 King Abdullah University of Science and Technology, Biological and Environmental Science and Engineering Division, Thuwal, Makkah, Saudi Arabia.; 5 Universidade de São Paulo, Instituto Oceanográfico, São Paulo, SP, Brasil.

**Keywords:** Curcumina, Curcuma longa, Microbiota intestinal, Hemodiálise, Doença Renal Crônica

## Abstract

**Introdução:**

O impacto da curcumina na microbiota intestinal de pacientes com doença renal crônica (DRC) não é bem conhecido. O objetivo deste estudo foi avaliar o efeito da *Curcuma longa L.* na microbiota intestinal de pacientes com DRC submetidos à hemodiálise (HD).

**Métodos:**

Análise secundária de dados provenientes de um ensaio clínico randomizado, duplo-cego e controlado por placebo. Os pacientes receberam 100 mL de suco de laranja, 12 gramas de cenoura e 2,5 gramas de *Curcuma longa L.*, três vezes por semana, após a sessão de HD (grupo curcumina), ou o mesmo suco sem adição de curcumina (grupo controle) durante 12 semanas. A composição da microbiota fecal foi estimada utilizando sequenciamento de leitura curta da região V4 do gene 16S rRNA na plataforma Illumina.

**Resultados:**

Onze pacientes participaram deste estudo, cinco no grupo curcumina (66,7% homens, 59 ± 16,7 anos, tempo médio de hemodiálise de 97 ± 62,6 meses, IMC 25,3 ± 2,9 kg/m^2^) e seis no grupo controle (60% homens, 57,5 ± 12,5 anos, tempo médio de hemodiálise de 48,3 ± 32,2 meses, IMC 25,2 ± 3,1 kg/m^2^). A suplementação com extrato de *Curcuma longa L.* não alterou a biodiversidade alfa nem a composição taxonômica dos indivíduos nos níveis de filo, família e gênero.

**Conclusão:**

A suplementação com 2,5 g de extrato de *Curcuma longa L.*, três vezes por semana durante 12 semanas, mostrou-se ineficaz na modulação da microbiota intestinal de pacientes com DRC em HD. Esses resultados devem ser interpretados levandose em consideração o pequeno tamanho da amostra, sendo recomendada a realização de estudos futuros com coortes maiores.

## Introdução

A disbiose intestinal consiste no desequilíbrio entre as bactérias intestinais, comprometendo, consequentemente, sua atividade metabólica, e essa condição é comumente encontrada em pacientes com doença renal crônica (DRC)^1 2 3^. Estudos experimentais e clínicos demonstraram que pacientes com DRC apresentam disbiose intestinal com elevada produção de metabólitos tóxicos por patobiontes e baixa abundância de espécies benéficas envolvidas na produção de ácidos graxos de cadeia curta^4,5^.

A perda da função renal está associada ao aumento da secreção de ureia no trato gastrointestinal e à geração de grandes quantidades de amônia a partir da hidrólise da ureia pelas bactérias intestinais^6^. O acúmulo de amônia aumenta o pH intestinal, causa irritação grave na mucosa, afeta o crescimento das bactérias comensais e mantém o estado disbiótico^6,7^.

Além disso, pacientes com DRC consomem menos fibras, o que, juntamente com o uso de quelantes de fosfato, reduz o tempo de trânsito colônico na microbiota intestinal^8^. Consequentemente, os solutos urêmicos produzidos durante o processo de fermentação pela microbiota intestinal se acumulam em pacientes com DRC^1,7^.

A disbiose intestinal em pacientes com DRC acelera a progressão da doença, causa complicações e inflamação sistêmica e aumenta as taxas de mortalidade. Portanto, estratégias nutricionais têm sido propostas para abordar a disbiose intestinal nesses pacientes^9,10^. A *Curcuma longa L.* é rica em compostos bioativos chamados curcuminoides e tem sido alvo de estudos recentes com pacientes portadores de DRC, demonstrando propriedades anti-inflamatórias cruciais^11,12^ e efeitos benéficos na saúde intestinal^13,14^.

A hipótese do presente estudo piloto foi de que a suplementação com extrato de *Curcuma longa L.* (95% de curcumina) modula a microbiota de pacientes com DRC submetidos à hemodiálise. Este estudo teve como objetivo avaliar o efeito desse extrato no perfil da microbiota intestinal de pacientes em hemodiálise (HD).

## Métodos

Foi realizada uma análise secundária de dados provenientes de uma pesquisa longitudinal, randomizada e duplo-cega em pacientes submetidos à HD^11^. Não foi realizado nenhum cálculo formal do tamanho da amostra para os desfechos avaliados nesta subanálise. O estudo foi aprovado pelo Comitê de Ética em Pesquisa da Faculdade de Medicina da UFF, registrado sob o número 2.346.933, bem como no site ClinicalTrials.gov, com o identificador NCT 03475017.

### Sujeitos

Foram incluídos no estudo pacientes com idade igual ou superior a 18 anos e submetidos à HD (por meio de fístula arteriovenosa) por mais de seis meses. Os critérios de exclusão foram gestantes, fumantes, pacientes que haviam feito uso de antibióticos nos três meses anteriores, indivíduos que utilizavam suplementos antioxidantes ou que consumiam açafrão regularmente e aqueles com doenças autoimunes e infecciosas, câncer, doenças hepáticas e AIDS. Os medicamentos utilizados antes do estudo foram mantidos durante todo o período da pesquisa.

### Desenho Experimental

Os pacientes foram randomizados de forma duplocega em dois grupos: o grupo da curcumina, que recebeu 100 mL de suco de laranja com 12 gramas de cenoura e 2,5 gramas de açafrão (95% de curcumina) três vezes por semana, após a sessão de HD, durante 12 semanas; e o grupo controle, que recebeu o mesmo suco sem curcumina.

O suco de laranja e cenoura utilizado como veículo para a administração da curcumina foi padronizado, e sua composição nutricional foi descrita anteriormente por Alvarenga et al^11^. Cada porção de 100 mL continha aproximadamente 98 kcal, 22,6 g de carboidratos, 0,2 g de lipídios, 1,0 g de proteína, 3,6 g de fibras, 38,6 mg de sódio, 44,0 mg de fósforo e 323,0 mg de potássio^11^. O extrato de *Curcuma longa L.* utilizado neste estudo foi previamente analisado por Alvarenga et al.^15^ por meio de HPLC-DAD. O extrato continha 75,31 ± 2,0% de curcumina, 18,42 ± 0,6% de demetoxicurcumina e 5,19 ± 0,2% de bisdemetoxicurcumina, totalizando 98,92 ± 2,8% p/p de curcuminoides totais^15^.

### Análise da IngestãO Alimentar e Avaliação do IMC

O consumo alimentar foi avaliado antes e após a intervenção utilizando o método de recordatório alimentar de 24 horas e calculado por meio do software *NutWin* ^®^ (Alvarenga et al.^11^). O índice de massa corporal (IMC) foi calculado como peso corporal (kg) dividido pela altura ao quadrado (m^2^).

### Coleta de Sangue e Análise Bioquímica

Exames bioquímicos de rotina, como albumina, fósforo, cálcio, potássio, ureia, paratormônio (PTH) e hemoglobina, foram obtidos a partir de prontuários médicos. Os níveis séricos de proteína C reativa de alta sensibilidade (hs-CRP), colesterol total, triglicerídeos e c-HDL foram determinados utilizando o Analisador Bioquímico *Bioclin* BS-120. O colesterol de lipoproteína de baixa densidade (LDL) foi calculado pela equação de *Friedewald*.

### Sequenciamento e Análise da Microbiota Intestinal

Para a análise da microbiota intestinal, os pacientes receberam tubos esterilizados para coleta de fezes e instruções detalhadas sobre o procedimento de coleta. A extração total de DNA foi realizada utilizando o kit *Quick-DNA Fecal/Soil Microbe DNA Miniprep* da *Zymo Research*, seguindo as instruções do fabricante. A qualidade e a quantidade do DNA foram determinadas por quantificação espectrofotométrica utilizando o *NanoDrop 2000* (*Thermo Fisher Scientific*)^16^.

O gene 16S rRNA (região V4) foi amplificado por PCR utilizando os *primers* universais 515F (5’-GTGYCAGCMGCCGCGGTAA-3’) e 806R (5’-GGACTACNVGGGTWTCTAAT-3’), acrescidos de etiquetas Illumina universais. Para gerar os amplicons, foi realizada uma termociclagem de 3 minutos de desnaturação inicial a 94 °C, seguida por 32 ciclos de 45 segundos a 94 °C, 1 minuto a 50 °C e 90 segundos a 72 °C, com uma etapa final de extensão de 10 minutos a 72 °C. Os amplicons resultantes foram então identificados com código de barras, agrupados e sequenciados com a plataforma Illumina NovaSeq PE250, seguindo as instruções do fabricante, na Novogene (Califórnia, EUA). No total, 22 amostras de pacientes em HD foram sequenciadas por amplicon.

As leituras de sequenciamento do amplicon do gene 16S rRNA foram pré-processadas com o uso do *pipeline* USEARCH (v.11) (Edgar, 2010^17^). As sequências resultantes foram *denoised* em Unidades Taxonômicas Operacionais de Raio Zero (zOTUs), que incluíram o agrupamento de amostras, ao mesmo tempo em que mantiveram etiquetas de amostras exclusivas nos cabeçalhos dos arquivos *fastq*, seguido pela montagem/fusão de leituras pareadas, remoção de *primers* e filtragem de qualidade. Além disso, as abundâncias foram calculadas para sequências únicas e agrupamento de zOTUs com 97% de identidade, seguido por uma etapa de redução de ruído (*denoising*) para a filtragem de quimeras, a fim de obter uma tabela de zOTUs. As tabelas de características, taxonomia e metadados foram exportadas como objetos *phyloseq* para análise subsequente no RStudio (v. 2023.06.0+421).

Para permitir a comparação em igualdade de condições entre os grupos estudados, os dados foram rarefeitos para análises comparativas posteriores. Os dados rarefeitos geraram índices de diversidade alfa (número observado de zOTU e diversidade de *Shannon*). Os dados brutos de zOTU foram normalizados com o pacote DESeq2 e utilizados para calcular a distância Unifrac ponderada utilizando o pacote *phyloseq* implementado em R. A análise de variância multivariada permutacional (PERMANOVA) foi realizada na matriz de dados para comparar a estrutura ou composição das comunidades microbianas. A análise multivariada permutacional de dispersão (PERMDISP) também foi feita utilizando a função *betadisper* (implementada no *vegan* 2.6-4) com 999 permutações. O pacote DESeq2 foi empregado para avaliar as abundâncias relativas diferenciais de leitura entre os grupos de amostras. Os gráficos e diagramas foram gerados com o pacote ggplot2 (v.3.4.0).

### Análises Estatísticas

O teste de *Shapiro-Wilk* foi utilizado para verificar a distribuição das variáveis da amostra. Para comparações entre grupos, o teste t de *Student* foi empregado para amostras com distribuição simétrica e o teste de *Mann-Whitney* foi utilizado para variáveis com distribuição assimétrica. Para comparações antes e depois dentro de um mesmo grupo, utilizou-se o teste t de *Student* pareado para variáveis com distribuição simétrica e o teste de *Wilcoxon* para variáveis com distribuição assimétrica. O nível de significância foi definido como inferior a 5% (p < 0,05). Os dados das características da amostra são expressos como frequências absolutas para variáveis categóricas e média (desvio padrão) ou mediana (intervalo interquartil) para variáveis quantitativas. As análises estatísticas foram feitas utilizando o software SPSS 23.0 (SPSS, Inc., Chicago, IL, EUA).

## Resultados

Um total de 11 indivíduos foi incluído neste estudo (Figura 1) (58,3% do sexo masculino; 58,2 ± 14,1 anos de idade; K/tV 1,4 ± 0,1; tempo em hemodiálise de 70,4 ± 52,3 meses e IMC de 25,3 ± 2,9 kg/m^2^). Os participantes foram divididos em dois grupos: cinco no grupo curcumina (66,7% do sexo masculino; 59 ± 16,7 anos de idade; tempo em HD de 97 ± 62,6 meses; IMC 25,3 ± 2,9 kg/m^2^) e seis no grupo controle (60% do sexo masculino; 57,5 ± 12,5 anos de idade; tempo em HD de 48,3 ± 32,2 meses; IMC 25,2 ± 3,1 kg/m^2^).

**Figura 1.** Fluxograma CONSORT do estudo.

Os parâmetros bioquímicos e as características da ingestão alimentar antes e após a intervenção são apresentados na Tabela 1. No início do estudo, não foram observadas diferenças estatisticamente significativas entre os grupos curcumina e controle em nenhuma das variáveis analisadas. No grupo curcumina, observou-se uma redução significativa no colesterol total, HDL e LDL. No grupo controle, também houve uma redução significativa no LDL, juntamente com um aumento significativo nos níveis séricos de cálcio e PTH.

**Tabela 1:** Características basais dos pacientes com DRC submetidos à Hemodiálise

Parâmetros	Geral (n = 11)	Curcumina antes (n = 5)	Curcumina após (n = 5)	Valores de p^a^	Controle antes (n = 6)	Controle após (n = 6)	Valores de p^b^	Valores de p^c^	Valores de p^d^
Exames de rotina	
CT (mg/dL)	161,9 ± 32,4	164,6 ± 25,3	139,6 ± 19,8	**0,01**	159,7 ± 39,7	158,4 ± 54,2	0,06	0,82	0,45
Triglicerídeos (mg/dL)	127,2 ± 49,2	104,8 ± 30,7	115 ± 55,7	0,19	145,8 ± 56,4	188,6 ± 121,7	0,06	0,18	0,21
HDL (mg/dL)	34,3 ±10,1	42,4 ± 7,4	39 ± 8,2	0,06	27,6 ± 6,4	37 ± 8,1	0,67	0,08	0,69
LDL (mg/dL)	102,4 ± 29,5	102,2 ± 28,9	77,6 ± 17	**0,01**	104,4 ± 32,6	83,7 ± 54,5	**0,01**	0,91	0,80
Cálcio (mg/dL)	8,9 ± 0,6	8,8 ± 0,4	8,6 ± 0,6	0,37	8,1 ± 0,7	8,7 ± 0,6	**0,01**	0,92	0,68
Fósforo (mg/dL)	4,8 ± 0,8	4,5 ± 0,7	5,1 ± 0,7	0,28	5,2 ± 0,9	5,7 ± 0,9	0,08	0,20	0,27
Albumina (mg/dL)	3,6 ± 0,2	3,6 ± 0,1	3,6 ± 0,2	0,53	3,6 ± 0,3	3,7 ± 0,2	0,54	1,00	0,45
Potássio (mg/dL)	4,7 ± 0,3	4,8 ± 0,2	4,9 ± 0,2	0,54	4,6 ± 0,5	4,7 ± 0,3	0,49	0,70	0,10
Hemoglobina (mg/dL)	11,06 ± 1,2	11,6 ± 2,4	10,9 ± 2,1	0,26	10,5 ± 1,4	11,8 ± 2,4	0,06	0,35	0,52
PTH (mg/dL)	560 (435,7-1414,8)	1027,5 (460,6-1856)	1075 (341-1846)	0,07	432,5 (208,3-1599,6)	899 (269-2640)	**0,01**	0,24	0,73
hsCRP (mg/L)	3,4 (2,5-11,5)	5,8 (1,4-13,1)	4,7 (0,2-16,6)	0,66	5,5 (0,1-16,2)	2,8 (0,7-6,7)	0,58	0,58	0,56
Ingestão alimentar	
Energia (Kcal/kg/dia)	22,1 ± 6,9	21,3 ± 8,6	21,1 ± 11,8	0,16	22,8 ± 6,0	23,1 ± 9,7	0,34	0,74	0,76
Carboidrato (g/dia)	220 ± 74,4	111,6 ± 67,8	211,5 ± 99	0,33	227,2 ± 85,1	274,7 ± 146,5	0,47	0,75	0,41
Proteína (g/kg/dia)	1,0 ± 0,4	0,9 ± 0,5	1,1 ± 0,6	0,17	1,05 ± 0,3	1,1 ± 0,6	0,27	0,76	0,96
Lipídio (g/dia)	34,7 ± 12,9	30,6 ± 17	24,7 ±11,4	0,12	38,0 ± 7	31,4 ± 18,3	0,78	0,37	0,48
Fósforo (mg/day)	923,8 (715-1242,1)	819,6 (316,6-1323)	779 (323-1444)	0,34	1201 (732-1490)	1169 (602-1956)	0,15	0,24	0,19
Potássio (mg/day)	1627 (1373-2492)	1529 (1030-2028)	1663 (456-2669)	**0,03**	1841 (1241-3296)	1730 (878-2432)	0,53	0,17	0,88

Abreviaturas - CT: colesterol total; PTH: paratormônio; HDL: lipoproteína de alta densidade; LDL: lipoproteína de baixa densidade; hsCRP: proteína C reativa de alta sensibilidade.

Notas - Os dados são apresentados como média (desvio padrão) ou mediana (intervalo interquartil) para variáveis quantitativas.

^a^Comparação antes e depois no grupo curcumina.

^b^Comparação antes e depois no grupo controle.

^c^Comparação basal entre os grupos.

^d^Comparação entre os grupos no final da intervenção.

Não houve diferença estatística na biodiversidade da microbiota intestinal dos pacientes em HD, medida pelo índice de Shannon (Figura 2A) e espécies observadas (contagem de OTU) (Figura 2B) entre os grupos (controle e curcumina) e os períodos de intervenção (pré e pós). Além disso, não foi observada diferença na composição taxonômica entre os grupos em nível de filo (Figura 3A), família (Figura 3B) e gênero (Figura 3C).

**Figura 2.** Diferença na biodiversidade antes e após a intervenção com curcumina. A diversidade ecológica bacteriana foi avaliada entre o grupo controle (azul) e o grupo curcumina (amarelo). (A) Análise da diversidade alfa com o índice de Shannon. (B) Análise da diversidade alfa com espécies observadas (contagem de OTU).

**Figura 3.** Composição taxonômica dos indivíduos nos grupos controle e curcumina, antes e após a intervenção. Gráficos de barras da abundância relativa microbiana média em nível de filo (A), família (B) e gênero (C) para indivíduos no grupo controle antes (Pré) e após (Pós) a intervenção e indivíduos no grupo curcumina antes (Pré) e após a intervenção (Pós).

## Discussão

No presente estudo, a suplementação com curcumina não alterou a diversidade nem a estrutura taxonômica da microbiota intestinal ao longo dasp 12 semanas de estudo em pacientes com DRC submetidos à HD. Este é o primeiro estudo a analisar o efeito da curcumina na composição da microbiota intestinal em pacientes em HD.

Em um estudo envolvendo pacientes com DRC não submetidos à diálise, a suplementação com 1000 mg de extrato de *Curcuma longa L.* por 6 meses modulou de forma eficaz a microbiota intestinal ao promover a redução de *Escherichia-Shigella* e o aumento de *Lachnoclostridium*. No nível de família, as espécies *Lactobacillaceae* aumentaram após 3 meses de suplementação^13^. Outros estudos demonstraram a eficácia da suplementação com extrato de *Curcuma longa L.* na redução de toxinas urêmicas produzidas pela microbiota intestinal em pacientes com DRC; entretanto, o papel da curcumina na influência da microbiota intestinal ainda não foi investigado. Salarolli et al*.* ^14^, em um ensaio clínico randomizado e duplo-cego envolvendo 28 pacientes em HD, constataram que a suplementação com 100 mL de suco de laranja contendo 12 g de cenoura e 2,5 g de açafrão, três vezes por semana durante três meses, reduziu as concentrações plasmáticas de p-cresil sulfato (pCS). Reis et al.^18^ demonstraram que a suplementação com 1500 mg de extrato de *Curcuma longa L.* durante doze semanas indicou uma tendência à redução dos níveis plasmáticos de pCS (p = 0,07) em pacientes em diálise peritoneal.

Os curcuminoides, incluindo a curcumina, podem ser metabolizados pela microbiota intestinal^18^através de um processo proposto de reação de hidroxilação, uma etapa metabólica microbiana comum, resultando na formação dos metabólitos hidroxicurcumina e dihidrocurcumina, que modulam a microbiota intestinal^19,20^. Além disso, foram identificadas outras vias metabólicas, demonstrando que a curcumina pode ser metabolizada pela microbiota intestinal por meio de processos de redução, metilação, desmetoxilação e acetilação, resultando na formação de tetrahidrocurcumina, ácido dihidroferúlico e 1-(4-hidroxi-3-metoxifenil)-2-propanol^20,21^. As bactérias provavelmente envolvidas na biotransformação e degradação do composto incluem *E. coli*, *E. fergusonii*, *Blautia* sp*.*, *Bifidobacterium*, *Lactobacillus*, *Enterococcus faecalis*, *Pichia anomala*, e *Bacillus megaterium* ^20,21,22^.

Portanto, a microbiota intestinal exerce uma influência significativa na biotransformação da curcumina, e seus metabólitos produzidos pela microbiota intestinal também desempenham um papel promissor na prevenção ou no tratamento de muitas doenças^22^. No entanto, dependendo de sua formulação, pode não ocorrer uma maior degradação microbiana dos compostos originais^19^. A formulação fosfolipídica leva a uma maior degradação microbiana dos compostos originais do que o extrato natural de curcumina. Em outras palavras, o processo de biotransformação microbiana é mais eficiente do que com o extrato simples de curcuminoide^19^. Isso fornece uma perspectiva sobre a formulação ideal de curcumina para a modulação da microbiota intestinal. Além disso, diferenças individuais na composição da microbiota podem causar diferentes biotransformações da curcumina^22^, o que pode interferir em seus benefícios. Esse fator pode transcender os achados do presente estudo.

A composição da microbiota intestinal é influenciada por múltiplos fatores, incluindo dieta, uso de medicamentos, estresse, atividade física e exposições ambientais. No presente estudo, a ingestão alimentar foi avaliada e considerada na interpretação dos resultados referentes à microbiota, em consonância com as evidências crescentes de que a dieta é um fator determinante na composição da microbiota, especialmente em pacientes com DRC^23^. No entanto, os demais fatores não foram avaliados, e sua possível influência na variabilidade microbiana intestinal não pode ser descartada. Conforme destacado por Van Hul et al*.* ^24^, definir um “microbioma saudável” continua sendo um desafio complexo devido à interação entre hospedeiro, estilo de vida e fatores ambientais.

Embora o foco principal deste estudo tenha sido a microbiota intestinal, análises secundárias demonstraram uma redução significativa nos níveis de colesterol total e LDL em pacientes que receberam curcumina. Esses resultados estão de acordo com evidências prévias que indicam os efeitos hipolipemiantes do extrato de *Curcuma longa L*. Alvarenga et al.^15^ relataram uma tendência à redução dos triglicerídeos em pacientes em HD sob suplementação com curcumina, corroborando seu potencial como abordagem adjuvante no manejo do metabolismo lipídico. Os mecanismos podem incluir a redução da absorção intestinal de colesterol por meio da inibição do transportador *Niemann-Pick C1-like 1* (NPC1L1), o aumento dos receptores de LDL no fígado e a regulação das enzimas envolvidas na síntese de lipídios, como a 3-hidroxi-3-metilglutaril-CoA (HMG-CoA) redutase e a sintase de ácidos graxos^25,26^.

Os achados deste estudo piloto devem ser interpretados à luz de algumas limitações, particularmente o pequeno tamanho da amostra, que limita substancialmente o poder estatístico para detectar diferenças na microbiota. Portanto, os resultados devem ser considerados exploratórios e geradores de hipóteses. Embora o extrato de curcumina utilizado neste estudo tenha sido padronizado para 95% de curcuminoides – uma formulação comumente empregada para garantir consistência e melhorar a biodisponibilidade em comparação ao açafrão bruto -, a absorção sistêmica limitada da curcumina ainda representa uma restrição reconhecida. Nesse sentido, estudos futuros podem se beneficiar do uso de sistemas de liberação mais avançados, como a coadministração com piperina, complexos fosfolipídicos ou formas nanoparticuladas, que demonstraram perfis farmacocinéticos superiores.

A ausência de modulação detectável da microbiota pode decorrer da complexidade do ecossistema intestinal e da duração relativamente curta do estudo (12 semanas), que pode ter sido insuficiente para revelar os efeitos da curcumina na microbiota. Embora o sequenciamento do amplicon do rRNA 16S forneça informações sobre os membros dominantes da comunidade microbiana e sua resolução taxonômica, ele oferece informações limitadas sobre os níveis taxonômicos inferiores. Esses níveis podem incluir espécies raras ou membros da comunidade cujas variações, embora significativas, podem não ser capturadas por meio desse método. Estudos futuros devem considerar o uso do sequenciamento metagenômico *shotgun*, que permite uma resolução taxonômica mais profunda e uma avaliação direta das vias microbianas funcionais, superando, assim, as limitações do perfilamento por rRNA 16S. Além disso, estudos futuros podem se beneficiar dos resultados em longo prazo de diferentes dosagens de curcumina na microbiota e explorar seus efeitos em desfechos mais amplos da DRC, incluindo saúde cardiovascular, função renal e qualidade de vida dos pacientes.

## Conclusão

A suplementação com 2,5 g de extrato de *Curcuma longa L.* (95% de curcumina) três vezes por semana durante 12 semanas mostrou-se ineficaz na modulação da microbiota intestinal de pacientes com DRC em HD. Incentiva-se a realização de estudos futuros com coortes maiores para explorar de forma mais aprofundada os efeitos potenciais da curcumina na microbiota intestinal desta população.

## Disponibilidade de Dados

O conjunto completo de dados que sustenta os achados deste estudo está disponível mediante solicitação à autora correspondente, Dra. Livia Alvarenga. O conjunto de dados não está disponível publicamente devido a questões éticas.
